# Balancing the use of language to enable care: a qualitative study of oral and written language used in assessments and allocations of community healthcare services for persons with dementia

**DOI:** 10.1186/s12913-016-1659-0

**Published:** 2016-08-16

**Authors:** Anette Hansen, Solveig Hauge, Ådel Bergland

**Affiliations:** 1 Faculty of Health and Social Sciences and Centre for Care Research, University College of Southeast Norway, Postbox 235, , 3603 Kongsberg, Norway; 2 Institute of Health and Society, Department of Nursing Science and Lovisenberg Diaconal University College, University of Oslo, Lovisenberggaten 15b, 0456 Oslo, Norway

**Keywords:** Community healthcare services, Healthcare assessment, Home care, Purchasers, Communicating sensitive topics, Dementia care, Alzheimer’s disease

## Abstract

**Background:**

Although a large number of people are diagnosed with dementia each year, the syndrome is still perceived as a sensitive and tabooed topic. Communication about dementia to those living with the syndrome and their relatives is often experienced as challenging by health professionals.

Failure to communicate clearly may threaten assessment and allocation of appropriate, effective healthcare services. Accordingly, the aim of this study was to explore how purchasers, assessing and allocating healthcare services to home-dwelling older people with dementia, described challenges in communicating about dementia with those with the syndrome and their relatives. Furthermore, the study aimed to explore the purchasers’ justifications for their choice of words.

**Methods:**

A qualitative study was conducted to investigate two data sources: focus group interviews with purchasers assessing need for healthcare services, and a review of administrative decisions written by those allocating services. Focus group data were explored using an interpretive approach and qualitative content analysis was carried out with the administrative decisions.

**Results:**

The purchasers found it challenging to talk and write about dementia to those with the syndrome and their relatives when assessing and allocating services. The purchasers were flexible in their communication and aimed to be open when talking and writing about dementia. However, euphemisms and omission were used extensively. Four justifications for the chosen verbal and written language were identified: avoiding disclosure; protecting the person with dementia; protecting the relatives/avoiding conflict; and last, taboo and stigma.

**Conclusions:**

Despite purchasers experiencing difficulties in communicating about dementia to those with the syndrome and their relatives, they did manage to communicate in a conscious and flexible way. The purchasers had several justifications for their language choice. However, extensive use of euphemisms and omission might threaten appropriate identification of needs and provision of high quality healthcare services. The challenges experienced by the purchasers demonstrate the need to focus on appropriate and flexible strategies for individually-tailored communication about dementia with people living with the syndrome.

**Electronic supplementary material:**

The online version of this article (doi:10.1186/s12913-016-1659-0) contains supplementary material, which is available to authorized users.

## Background

This study investigates the challenges faced by purchasers assessing and allocating healthcare services for home-dwelling older people with dementia (PWDs) when talking or writing about dementia. The data were collected within a larger study investigating how healthcare workers in four Norwegian municipalities emphasize and take care of the psychosocial needs of home-dwelling PWDs.

Worldwide, over 46 million people live with dementia [1]. The word dementia originates from the Latin “de mens”, which translates as “no mind” [2]. Colloquially, various pejorative terms for dementia have been used, including “crazy”, “insane”, and “old age stupor” [3]. To change negative associations and reduce stigmatization, Harris and Keady [4] advocate associating dementia with more positive terms. It has also been argued that the term dementia should no longer be used because of established negative associations and because it does not constitute a definitive medical diagnosis [4]. In an attempt to reduce stigmatization, the Japanese government decided in 2004 to change “ChihÕ”, the Japanese word for dementia, to a less negative term “NinchishÕ”, meaning disease of cognition [5]. PWDs have also objected to the use of the terms dementia and Alzheimer’s disease in favour of more descriptive terms, such as “memory loss” or “forgetfulness” [6]. However, some PWDs wish to receive a specific diagnosis [7].

Dementia is reported to be one of the most feared diagnoses among older adults [8]. The fact that dementia is incurable and leads to reduction in an individual’s cognitive capacity are factors that may contribute to fear, shame and stigmatization [9]. Cognitive impairment affects ability of PWDs to remember, recognise and relate to their loved ones. Such cognitive changes cause increased dependency and, in many cases a more restricted social life [9] owing to shame and a wish to conceal cognitive symptoms. A lack of awareness and understanding of dementia and its trajectory may result in further stigmatization, and barriers to diagnosis and care [5]. This can also cause difficulties talking about dementia. The perception of and language used to talk about dementia not only affect PWDs but also their families, health professionals, policy makers, dementia researchers, and the general public [10, 11].

Communication about dementia has been addressed in previous research [12], often in relation to physician–patient conversations regarding disclosure of the diagnosis [13–17]. Physicians frequently experienced great difficulty in finding appropriate ways to talk with the patients about dementia and disclose the diagnosis [18, 19]. Many physicians wanted to disclose the diagnosis to patients, but were reluctant to use words like dementia and Alzheimer’s disease. They preferred vaguer terms and euphemisms [17, 20, 21]. Despite the intention of some physicians to be direct and use medical terms, euphemistic terms were often used, such as memory problems and thinking problems [19].

To our knowledge, little research has been conducted into the experiences of purchasers regarding how to talk about dementia, and their choice of words in charting conversations and administrative decisions.

## Methods

### Aim

First, the study aimed to explore how purchasers, who assess and allocate healthcare services to home-dwelling older PWDs, described the challenges regarding how to talk and write about dementia. Second, the study aimed to explore the purchasers’ reasons for their choice of words.

### Design

As little was known about this topic, the study had a qualitative, descriptive multi-site design [22].

### Study setting

In Norway, there are currently approximately 78,000 PWDs [23], and more than 50% of these live in their own homes. Each municipality is responsible for providing appropriate healthcare services to home-dwelling PWDs [24]. To support healthcare services in large- and medium-sized Norwegian municipalities, a purchaser-provider split model is often used [25]. This model separates administration and provision of healthcare services into two separate units: a purchaser and a provider [26]. The purchasers assess the needs of PWDs and allocate services accordingly. This is often done in three steps. When the purchaser unit receives an application for healthcare services, they first obtain information about the person with dementia from their physician, other health professionals or relatives. The second step of the assessment is conducted as a charting conversation in the home of the person with dementia, usually with family members present. In this conversation, the person with dementia’s resources and needs are identified. Finally, the purchaser forms an administrative decision stating which services the person with dementia shall receive and the reasons for receiving them [27]. The administrative decision is sent to the providers of healthcare services and the person with dementia. The providers (e.g., home nursing and day care centres) act based on the decision of the purchaser.

### Recruitment and inclusion criteria

This study was conducted within healthcare services in four medium-sized Norwegian municipalities, all comprising both urban and rural areas. Recruitment was conducted in two steps. First, municipalities were recruited. An invitation to participate was emailed to healthcare service leaders in 13 medium-sized municipalities in Eastern Norway, each with more than 30,000 citizens (to ensure a sufficient number of administrative decisions). We included the first four municipalities to respond positively. In the second step, leaders provided administrative decisions and recruited purchasers for focus group interviews.

Administrative decisions were included where they allocated home nursing services (home nursing and domestic help) or day care centres to home-dwelling people over 70 years old, with dementia or with symptoms indicating dementia. Only those over 70 years were included as younger PWDs were often offered additional or different services. As a number of cases of dementia go undiagnosed [28, 29], the purchasers or the home nursing services had to observe symptoms consistent with dementia if a formal diagnosis had not been made. As the purchasers did not always have information about the specific type of dementia, we did not restrict inclusion based on type of dementia.

For inclusion in the focus group interviews, purchasers had to have extensive experience of assessing and allocating healthcare services to older home-dwelling PWDs. Since the purchasers assessed and allocated services for people with different health problems and in various age groups, the purchasers who had the most experience with assessing and allocating health care services to home-dwelling PWDs were recruited.

#### Participants in the focus group interviews

The four focus groups comprised four to six purchasers, with 19 purchasers in total (Table 1). All purchasers were female, aged 30 to 65 and with experience ranging from 8 to 40 years in various roles within different areas of the healthcare services. The purchasers were mainly registered nurses with or without additional specialized/post-bachelor education. However, social educators, occupational therapists, and physiotherapists also participated.

**Table 1:** Source of data

	Municipality A	Municipality B	Municipality C	Municipality D	Total
Focus group interview participants	4	6	5	4	19
Administrative decisions	59	99	58	30	246

### Data collection

Two types of data were collected: administrative decisions and focus group interviews. Data were collected between September 2012 and June 2013.

#### Administrative decisions

In accordance with the inclusion criteria, leaders of the purchaser units obtained administrative decisions and anonymized them by removing name, date of birth and address before delivery. Based on experience that administrative decisions usually contain limited descriptions of the causes and rationales for allocated services, we asked for 50 administrative decisions from each of the four municipalities to ensure sufficient data. We received a total of 268 administrative decisions, 246 of which were included (Table 1). Twenty-two decisions were not included, as they did not meet the inclusion criteria (e.g., granting for nursing home).

#### Focus group interviews

Semi-structured focus group interviews were used to explore further how psychosocial needs were assessed and allocated by the purchasers. The interviews also provided the purchasers an opportunity to elaborate on the administrative decisions in more detail. The focus group interviews were conducted from a descriptive perspective, allowing the participants to discuss and share their experiences and in-depth knowledge with each other, which led to rich and nuanced descriptions [30, 31].

The interview guide covered various aspects of assessing psychosocial health and needs with relatively open questions, allowing participants to focus on what they considered important [31–33]. Examples include: “Can you please describe how and what you consider and emphasize when you are in PWDs’ homes assessing their needs?”; “If we look more closely at psychosocial health, what do you do to identify related needs?”; and “In what way are psychosocial needs described in the administrative decisions?” (For complete interview guide se Additional file 1). The interview guide was adjusted after the first interview as we found it beneficial to use examples of administrative decisions as a starting point for the discussion. Therefore, in subsequent interviews we distributed two examples of administrative decisions from the interviewees’ own municipality and encouraged them to discuss these, providing in-depth clarification of what they emphasized when they made the decisions. During the focus group interviews, the purchasers responded to each other’s statements and reflections, stimulating each other to consider viewpoints they might not otherwise have thought of alone [30, 33, 34]. The first author conducted all focus group interviews. The second author participated in the first focus group interview as an assistant-moderator [33]. Immediately after each interview, the first author prepared a brief note containing “main experiences” and content that appeared important during the interview [33]. These notes were discussed with the other authors shortly after the interviews had been conducted. The focus group interviews lasted between 1.5 and 2 h and were held at the purchasers’ workplace at their request.

### Data analysis

Analysis of data did not occur linearly, instead an iterative approach was taken [32], which led to an unexpected outcome. Assessment of psychosocial needs and service allocation to meet these needs were the main topics in the interview guide. However, the purchasers’ reports of difficulties in talking and writing about dementia as the cause of healthcare needs were so prominent in the interview text that we could not ignore these statements [34–36]. The data from the focus group interviews were the primary source in the current study. Therefore, we began by analysing the focus group interviews to gain the purchasers’ descriptions of how they communicated about dementia in the charting conversations and what they emphasized when writing the administrative decisions. Subsequently, the first author analysed the 246 administrative decisions. Finally, the results of the two separate analyses were compared and assessed for similarities and differences.

#### Analysis of focus group interviews

The focus group interviews were audio-recorded and transcribed verbatim by the first author soon after each interview [30]. When the transcription was completed, the recordings were carefully listened to several times to ensure accuracy between the transcriptions and the audio recordings [30]. Confirmatory sounds, laughter and pauses in the talk were included in the transcriptions. This helped to ensure understanding among the second and third authors when reading parts of the transcriptions. The first author read the transcripts several times to ensure familiarity and gain an overall picture [30, 32].

The transcripts were then transferred into NVivo-10 software [37], which facilitated a systematic organization of the data. A preliminary understanding was developed by reading the transcripts more closely [30]. Meaningful units of the purchasers’ descriptions and explanations relevant to the study aim, or which seemed to have a specific meaning to the purchasers, were selected by the first author. Examples of meaning units could be: “Memory disease is a gentler way to write it” or “Shall we use ‘dementia’, ‘cognitive decline’ or ‘forgetful’?” Meaningful units were coded [30] and categories were created, including: ‘hidden to the PWD’, ‘afraid to offend’, ‘wish to be honest’. A search for similarities and differences between the categories was conducted, and overlapping categories were grouped together [32]. For example, the category ‘wish to be honest’ was grouped into the category ‘open’. Last, a holistic analysis and interpretation of the categories was performed.

During the analysis, new points of view and a holistic perspective were sought by returning regularly to the original interviews, both listening to the tapes and reading the transcripts. The first author held primary responsibility for conducting the analysis. However, there was a high degree of cooperation between the three authors throughout all steps of the analysis. Meaningful units, codes, and categories were developed, reviewed, and discussed at regular research meetings to ensure a common understanding of the data and to obtain a consensus at each stage of analysis.

#### Analysis of administrative decisions

The focus group interviews were the primary data source in the study. The purchasers had in the focus group interviews described what they emphasized when formulating administrative decisions and challenges experienced in describing reasons for the person’s healthcare needs. The administrative decisions provided a second data source to further explore these issues [36]. As such, the two analyses were linked thematically. A separate, independent analysis of the administrative decisions was also conducted.

Administrative decisions were subjected to a qualitative content analysis [38]. Each of the 246 administrative decisions was numbered and read several times. Meaningful units relevant to the study aim were extracted and categorised. Examples of meaningful units include: “You suffer from Alzheimer’s”; “You have reduced memory”; and “You have started to forget a little.” Meaningful units were grouped into three categories: ‘direct’, ‘refine’, and ‘dementia not mentioned’. Furthermore, the number of administrative decisions in each category were counted. This counting provided a valuable description of patterns in the data [35] and facilitated a description of the sample of the administrative decisions and any inequalities [39].

#### Analysis of findings from both data sources

Findings from the analysis of focus group interviews [35] and administrative decisions were further analysed together, to allow an overall interpretation. The categories from the administrative decisions were consistent with categories from the focus group interviews. For example, the category ‘open’, developed from the focus group interviews, was grouped together with the category ‘direct’ from the analysis of the administrative decisions. In this process, we developed three final categories. These were ‘direct language’ (e.g., Alzheimer’s disease, dementia), ‘use of euphemisms’ (e.g., forgetful), and ‘omissions’ (e.g., not mentioning dementia or related subjects). Combining data from the two analyse may provide a deeper insight into the study theme, compared with using a single data source [30].

## Results

Our findings highlight the challenges and complexity the purchasers experienced when talking and writing about dementia. The findings are reported below.

### Conscious and flexible choice of language

The purchasers talked about dementia diagnosis and related issues as a subject to be approached cautiously. It seems that they had developed an advanced practice for how they approached and talked about dementia. The purchasers explained that during a charting conversation they noticed reactions of PWDs or their relatives, and subsequently changed their wording. For example, they might ask PWDs about how they experienced their memory. If a PWD responded in an irritated tone that memory was not a problem, the purchasers would often choose to avoid the topic or talk about another subject until the memory theme was tried again. They might say, “It is common to forget a little with age; how is it with you?” The PWD might respond positively and describe his or her situation or continue to express a desire to avoid the topic. The purchasers explained that PWDs, and in some cases relatives, had to be protected from too direct speech. Cautiousness in using the words dementia or Alzheimer’s in charting conversations was similarly observed in the administrative decisions. Several purchasers explained that they had to be careful with what they wrote, but that healthcare professionals, using their expertise, could read between the lines. Three ways of communicating with PWDs about dementia in charting conversations and administrative decisions were identified: openness, euphemisms, and omission.

#### Openness

In the focus group interviews, some purchasers emphasized the importance of being direct in the charting conversation. One said: “I always try to be direct and ask a PWD how he or she experiences the situation.” This was the only description offered by the purchasers of how they tried to use direct communication. However, we would not consider this an example of truly direct communication and so no explicit descriptions of how purchasers managed to be direct in the charting conversations were obtained.

In relation to the administrative decisions, several explained that it was important to use the word ‘dementia’ or a precise diagnosis in these documents. Administrative decisions that mentioned dementia as a cause for allocated services may use the following wording: “You suffer from dementia, which is why you need help and facilitation in everyday life”. However, few administrative decisions were that direct (Table 2).

**Table 2:** Administrative decisions divided among the three categories

Openness	Euphemism	Omission	Total
34	102	110	246

#### Euphemisms

In charting conversations, euphemisms were often used. The purchasers explained that they had to “cover up” the message when the word “dementia” might be perceived as offensive or stigmatizing. The purchasers then approached PWDs with questions that indicated memory problems as a normal or natural part of aging. For example, they described that they asked questions like: “We all forget a little; how is it with you?” or “We remember a little worse when we get older. What do you think about it? Is this a problem for you?”

Euphemism was also often used in the administrative decisions (Table 2). The purchasers used correspondingly vague vocabulary like: “You are a little forgetful, so it is necessary for you to receive some help…” or “You have an illness that causes some memory problems”.

#### Omission

The third way of approaching the dementia diagnosis or related subjects in charting conversations was by omitting. The purchasers sensed that the word dementia was too loaded and so omitted it. One of the purchasers stated: “Sometimes you feel reluctance from a PWD, in which case you avoid the word entirely.” Another said: “It’s difficult to talk about the cognitive failure that you do not really want to say that they have.” When the purchasers avoided mentioning the dementia diagnosis and related issues, physical challenges were frequently used as a basis for all of the needs of PWDs.

Some purchasers regarded the administrative decisions as a justification for the municipality. Therefore, it was deemed sufficient if the need for healthcare, diagnosis, and related challenges formed part of the underlying assessment but were not included in the administrative decision. This was also evident in the analysis of administrative decisions (Table 2), where diagnosis or disabilities were often omitted. A typical example was: “When assessing your application, we based your needs on the charting conversation.” Reference to the underlying assessments, which were not visible to the PWD, allowed the actual cause to be avoided in the administrative decisions.

### Justifications for flexible language as a means to secure appropriate healthcare services

Regarding the charting conversations and the administrative decisions, the purchasers stressed that when allocating services they would ideally use diagnostic terms, such as “dementia” or “Alzheimer’s disease”. Despite this, they actually mostly used euphemisms or omissions. Four justifications for the chosen oral and written language were identified: avoiding disclosure; protecting PWDs; protecting the relatives/avoiding conflict; and last, taboo and stigma.

#### Avoiding disclosure

There were cases where the purchasers were unsure if the person with dementia had been previously assessed for or diagnosed with dementia. It might also be uncertain whether the physician had informed the person with dementia about the diagnosis. One said: “I have sometimes been unsure whether they know it or not. Have they received information? I’m afraid of telling them something they do not know.” This uncertainty led to a cautious approach to the subject, using euphemisms or omissions.

#### Protecting PWDs

The purchasers approached the dementia issue carefully to protect PWDs. “Words have the power to hurt,” they argued. Consequently, directness or full openness regarding the diagnosis and associated impairment could be too hard for PWDs to cope with. A purchaser explained: “We often have contact by telephone with the relatives, both before and after the charting conversation … what the relatives have to say can be perceived as very painful and difficult.” The purchasers were concerned about protecting PWDs and contacted relatives to avoid talking with PWDs about issues that might harm them. In this way, relatives could also provide information that they did not want to convey in the presence of the person with dementia.

In many cases PWDs’ lack of insight into their illness or if they had forgotten they had been diagnosed with dementia seemed to affect how the purchasers chose their words during charting conversations. The use of direct words, such as dementia or Alzheimer’s, could cause frustration or anger in PWD, which may result in refusal to accept help being offered. The purchasers often deliberately chose to be vague or to use euphemisms in their statements to secure acceptance of services by PWD.

The purchasers were also discreet when describing the dementia diagnosis in the administrative decisions. As a justification, they cited a desire to protect PWD against the strain of seeing the dementia diagnosis written down: “You weigh the information, since the person with dementia who gets the administrative decision can read it.” It was seen as more harmless for the person with dementia if they used euphemisms or omissions when they described the reason for the allocation of healthcare services. However, purchasers highlighted that needs had to be stated clearly, as: “If the cause of the need is not stated clearly in the administrative decision, PWD may not receive the help they are entitled to.”

#### Protecting relatives and avoiding conflict

During telephone contact prior to the charting conversation, the purchasers reported experiencing a denial or refusal to talk about dementia by some relatives. In some cases, this led to a total avoidance of the subject in the charting conversation, to protect the relatives. It also seemed that the purchasers avoided “dementia issues” in both the charting conversations and the administrative decisions if they thought it could lead to conflict between the PWD and their relatives. One expressed it this way: “…relatives become very anxious about what will happen between themselves and the person with dementia after we have left.” The fact that relatives most often had applied for services on behalf of the person with dementia could worsen this situation. One of the purchasers explained: “It can cause painful feelings to come to surface, and while the person with dementia himself denies it, relatives can be very insistent on how poorly they can manage, and then it becomes a difficult situation.”

#### Taboo and stigma

In the purchasers experience dementia is still associated with a taboo. One described a situation in which “…the relatives reject contact with friends because the illness is so tabooed.” Some purchasers reported that to reduce fear or stigma they chose to use words such as memory problems or forgetfulness, which the purchaser thought were not so stigmatizing. Several purchasers phoned relatives prior to the charting conversation to explore how open the family was about dementia: “Some people talk about it as though it is a completely unremarkable thing, just like an aching finger, for others it is the big elephant in the room…it fills the entire room, but no one talks about it. It is so dominant, but will not be materialized into words, although it is the essence of the whole situation.”

The family’s openness and understanding influenced how direct and open the purchasers could be in the charting conversation. Regarding the administrative decisions, many purchasers were reluctant to use words such as dementia and Alzheimer’s since these words were connected with considerable taboo and stigma. It was easier to be open regarding physical impairment than reduced cognition. One explained: “With dementia, many are very concerned about concealing their cognitive failure. They camouflage so much more than you would do if you had a physical handicap. It's different to say that I didn’t manage to get onto the bus because of a physical handicap, than saying I do not understand how to take the bus.” The purchasers related how taboo and stigma in relation to dementia affected PWDs’ willingness to be open about their condition. Two of the purchasers talked about the importance of greater transparency to counteract stigma in society: “There must be more openness in society in general about dementia. It is a condition that many get, so there shouldn’t be any taboo or hush-hush.”

### Overall interpretation: flexible use of language to ensure that PWD accept and receive help

The purchasers found it demanding to talk about dementia with people living with the syndrome, and took special consideration in relation to language used in the charting conversation and the administrative decisions. Overall, our interpretation indicates that the main goal for the purchasers was to ensure that PWDs accepted and received the help they needed and were entitled to. To achieve this, the purchasers exercised great awareness, with conscious assessment of which words they could use when talking and writing about dementia. A flexible alternation between euphemisms and omission in the same conversation was described, and one approach did not exclude the use of the other. It was evident that this alternation between different wordings was based on a continuous, conscious observation and interpretation of the responses from PWDs and their relatives. Despite a wish to be open, protecting PWDs from direct words was strongly emphasized to achieve the main goal: acceptance of and fulfilment of services.

## Discussion

Our findings indicate that purchasers find it challenging to talk with PWDs and their relatives about dementia and to use the dementia diagnosis in administrative decisions. The main goal for purchasers was to ensure that PWDs received the healthcare services to which they were entitled. To achieve this goal, purchasers exhibited advanced practice involving great flexibility while communicating about dementia, particularly in their use of euphemisms and omission.

### Dementia - a difficult issue to talk about

Despite various guidelines regarding talking about dementia [40–43], health professionals are struggling to find and agree on the most suitable words to use and ways to communicate about dementia with PWDs and their relatives. The challenges faced have been addressed in previous research, mainly regarding disclosure of a dementia diagnosis by physicians [15, 16, 18]. There is increasing support for openness about the syndrome [29], and the majority of people, either with or without cognitive impairment, would like to know if they were affected [7, 29, 44]. Interestingly, in contrast a large number of relatives prefer that the diagnosis be withheld from the PWD [16, 17, 45, 46].

Arguments for being open about dementia diagnoses are often related to PWDs’ right to know, to enhance autonomy, and to empower PWDs to participate in planning for their future [7, 29, 46, 47]. Consistent with our study, despite aiming for openness, euphemisms and omission are frequently used [15, 19]. In the context of disclosure, omission is often related to protecting PWDs from stigma [15, 20, 48]. Alternatively, it may be used owing to the physician’s lack of knowledge and communication skills [15, 43, 49], uncertainty about the diagnosis [16, 43, 46] or fear of negative reactions from PWDs [16, 43]. In the present study, these justifications were also given, although none of the purchasers described using euphemisms or omission owing to lack of skills. It is interesting that none of the purchasers mentioned factors such as lack of communication skills or feeling uncomfortable broaching the topic, as these could be present and have an impact on how they choose to communicate. Euphemisms or omission were also related to not knowing whether the PWD had received a diagnosis and fear of causing harm by telling them something they did not yet know. The purchasers’ worries may be well founded, as previous studies reveal that a significant number of physicians withhold the diagnosis from PWDs, despite best practice recommendations to provide the diagnosis in an informative and sensitive way [13, 43].

Since there is limited research regarding ways of talking about dementia with PWDs in a healthcare context, research within the context of disclosure is included in the discussion. Our findings bear many similarities to research related to disclosure. A major similarity is that both physicians and purchasers find it challenging to choose the “right words” and communicate about dementia with PWDs and their relatives [14, 19]. However, there are also differences between the two contexts. For example, a physician’s responsibility is to assess PWDs, make a diagnosis, disclose it, and eventually prescribe treatment. In contrast, when purchasers conduct assessments and allocations the diagnosis is often known and PWDs and their relatives might, to a certain extent, have adjusted to the diagnosis. Investigating the communication challenges in a novel context, this study contributes new knowledge and understanding of communication challenges faced by purchasers, and their influence on assessment and allocation of services.

### Impact of euphemisms and omissions on PWDs’ ability to participate in decision making

Recently, there has been considerable focus on empowering PWDs to be active participants in decisions about healthcare service provision [50]. To ensure high quality assessment and appropriate allocation, purchasers must provide necessary and sufficient information before an administrative decision is made [51]. Furthermore, as far as possible healthcare services should be planned in collaboration with PWDs [52]. The dementia syndrome involves several challenges owing to altered cognition, which affects the ability of PWDs to remember, have insight into their situation, make judgements, and state their needs [53]. This may affect PWDs’ capacity to participate in decision making. However, the extent to which PWDs have insight into their situation differs. Home-dwelling PWDs may often have some insight [54], which they can use to participate in, share or delegate decision making [55]. However, if they are to be enabled to participate, purchasers have to ensure that PWDs receive the information they need to do so. Communication must be adjusted to the capacity of each PWD. Norwegian legislation states that in dementia it may be necessary to involve relatives to contribute to and make decisions together with or on behalf of a PWD, but the recipient of the service should participate as much as possible [52]. Relatives and health professionals can provide a good picture of the PWD's resources and needs. However, in the charting conversation purchasers should, where possible, facilitate an interactive process between PWDs, their relatives, and themselves [15, 56].

Previous studies on disclosure of dementia have shown that in many cases relatives receive more direct and precise information than PWDs [13, 14, 46], and that omission and euphemisms might be requested by the family [19, 57]. This was also evident in the current study. Purchasers often contacted relatives prior to the charting conversation to obtain information about the PWD and to get the relative’s view on whether or not to talk about or mention the word dementia in the charting conversations. This indicates that in some situations, a PWD may not have the best information available to be an active participant, since part of the conversation does not include the person. Although some PWD may not get the opportunity to participate, research has shown that most commonly decision making is shared between PWD and their relatives [55]. To assess and allocate good healthcare services and enable PWD to participate, information provided must be sufficient and adjusted to the individual PWD's capacity, using understandable terms and language that clearly capture and describe the PWD's needs in a careful way [58]. In the present study, the focus on protecting PWD, by omission or use of euphemisms, was more prominent than ensuring that they were informed and able to participate in decision making. Although this “protection” of PWD had good intentions, it may not always be in their best interest. When purchasers avoid talking about the cause of PWDs’ needs, it may limit the ability of PWD to participate in decisions concerning future care and treatment [29].

### Being eager to protect - a threat of inadequate healthcare services

A major reason for not being direct and open in charting conversations and administrative decisions was the purchasers’ fear about PWDs’ reluctance to accept help if dementia is cited as the reason for the services. One might argue that knowing the reason for the allocated services may persuade PWDs to accept help [16], and that open and direct communication in the charting conversation provides PWDs with an opportunity to discuss their needs and wishes, thereby enabling the purchasers to allocate more tailored services. Open communication could also ensure that the situation is more comprehensible, manageable, and meaningful for PWDs and their relatives [59], as use of omission and euphemisms may lead to lack of clarity [17].

The use of omission and euphemisms may also result in vague assessments, which could adversely affect the quality of service [25]. It is possible that the purchasers’ eagerness to protect PWDs from directness limits the possibility that they receive help. Some purchasers explained that a consequence of using euphemisms or omission in the administrative decisions could be that PWDs did not receive the help to which they were entitled. A study of the perspectives of PWDs’ and caregivers’ on disclosure of dementia, reported that over 75% of PWD would like to actually read their diagnosis [7]. This indicates that the purchasers might be more flexible, considering not only the use of omission and euphemisms but also a more extensive use of openness. However, the same study reported that in some cases informal caregivers experienced that the PWD showed anger or denial when the words dementia or Alzheimer’s disease were mentioned [7]. These words tended to overshadow the discussion and hampered communication, resulting in poor understanding of what they were told during the disclosure [7]. Anger and denial could also be present in the charting conversations and purchasers had to exercise considerable flexibility in choosing which words to use, to avoid hampering communication. If they were too direct, PWDs might become closed off and show reluctance. The ideal was openness towards the PWD, but does this mean that one should always be precise and direct, using words like dementia or Alzheimer’s disease, or could euphemisms be considered to be “sufficiently” open?

The purchasers described the use of euphemisms as a tool to reduce fear and stigma. It is possible that the purchasers aided PWDs in camouflaging the syndrome by omitting or circumnavigating the directness of the terms dementia or Alzheimer’s, normalizing the communication using universal and general terms [49]. The report by the Nuffield Council on Bioethics about ethical issues in dementia states that stigma may be an obstacle to provision of high quality healthcare services [53]. Additionally, purchasers may unintentionally contribute to the maintenance of taboo and stigma associated with the syndrome by avoiding the topic.

Euphemisms and omissions may hamper communication, but use of euphemisms may also provide a way to clear a path for greater openness [47, 49] or to convey the message in a more acceptable and accessible manner [17]. Euphemisms allow PWDs to avoid talking about the dementia issue if they do not want to [49]. By using euphemisms, the purchasers can probe how open PWDs are regarding their condition and needs without offending or being confrontational, as well as providing them with an opportunity to be more direct and open if preferred. With this as a backdrop, euphemisms might be used as a reasonable starting point in the charting conversation. We do not claim that there is a clear answer on how best to communicate about dementia. However, the extensive use of omission might indicate that some PWDs may not receive appropriate, individually-tailored healthcare services since the main reason for provision of healthcare is not discussed with the PWD.

### Strengths and limitations

A strength of this study is that two different data sources were used: focus group interviews and administrative decisions. Another strength is that the same author conducted all focus group interviews, transcribed them, and reviewed all the administrative decisions. Furthermore, the data were discussed thoroughly among the three authors, providing a broader and more nuanced understanding. A limitation of the study is that only female purchasers participated, which may have influenced the findings. However, the sample is representative of these services, which are staffed mainly by women. Another limitation is that the findings cannot be generalized to all situations where purchasers assess and allocate healthcare services. Nonetheless, this study has identified that communication challenges exist during the assessment and allocation process.

### Further research

More research is needed to further explore purchasers’ communication about this sensitive topic with PWDs and their relatives. This would provide more insight and optimize the assessment and allocation of healthcare services. Future studies should also include observation of what actually happens during charting conversations with PWDs. Additionally, PWDs’ and relatives’ views on talking about dementia during assessment and healthcare allocation should be included in future research.

## Conclusions

Purchasers found it challenging when communicating about dementia with PWDs and their relatives. Despite this, thy managed to communicate in a conscious and flexible way. Purchasers provided several justifications for their language choice. However, their extensive use of euphemisms and omission in charting conversations, as well as administrative decisions, might limit proper identification of needs and provision of high quality healthcare services. The challenges experienced by purchasers may indicate a need for suitable and flexible strategies of individually-tailored communication about dementia with those living with the syndrome.

## Additional file

Additional file 1: Interview guide. (DOCX 14 kb)

## Abbreviation

PWD: Person with dementia
